# Integrative Bioinformatics Analysis Reveals COL13A1 and COL23A1 as Potential Diagnostic and Prognostic Biomarkers in Thyroid Cancer

**DOI:** 10.1002/hsr2.72617

**Published:** 2026-06-15

**Authors:** Md. Wahidul Islam, Md. Minhajur Rahman, Homaira Naznin, Md. Shohel Hossain, Tahmina Akter, Zayeda Akter Shatabde, Md. Jubayer Hossain

**Affiliations:** ^1^ Big Bioinformatics Lab Center for Health Innovation, Research, Action, and Learning—Bangladesh (CHIRAL Bangladesh) Dhaka Bangladesh; ^2^ Department of Microbiology Jagannath University Dhaka Bangladesh; ^3^ Department of Pharmacy Jahangirnagar University Dhaka Bangladesh; ^4^ Department of Microbiology Noakhali Science and Technology University Noakhali Bangladesh; ^5^ Center for Health Innovation, Research, Action, and Learning—Bangladesh (CHIRAL Bangladesh) Dhaka Bangladesh; ^6^ Department of Public Health Daffodil International University Dhaka Bangladesh

**Keywords:** bioinformatics analysis, cancer biology, COL13A1, COL23A1, precision medicine, thyroid carcinoma

## Abstract

**Introduction:**

Thyroid cancer, the most prevalent endocrine malignancy globally, poses challenges owing to the limited understanding of its molecular drivers. Previous research has highlighted collagen genes, such as COL13A1 and COL23A1, as key players in thyroid cancer. This study aimed to comprehensively investigate gene expression, genetic alterations, DNA methylation, and the prognostic significance of COL13A1 and COL23A1.

**Methods:**

This study employed a multi‐omics strategy leveraging the TCGA database and the following resources: TIMER2.0, GEPIA2, UALCAN, HPA, cBioPortal, STRING, Enrichr, Kaplan–Meier Plotter, and R programming.

**Results:**

We observed distinct expression patterns for COL13A1 and COL23A1. COL13A1 was significantly upregulated, while COL23A1 was downregulated in tumor tissues compared to normal tissues. Expression levels vary by sample type, tumor stage, and histology, with higher COL13A1 staining intensity and moderate COL23A1 staining observed in tumors. Both decreased COL13A1 and increased COL23A1 expression have been linked to poor prognosis. Promoter methylation levels also differ, showing higher COL23A1 methylation in tumors. SGIP1 and SLC26A4 were identified as the most co‐expressed genes. Functional validation using GEO datasets further supported the potential of COL13A1 and COL23A1 as prognostic markers in thyroid cancer.

**Conclusion:**

In thyroid cancer, COL13A1 and COL23A1 have emerged as diagnostic and prognostic markers. Co‐expression analysis suggests interactions between SGIP1 and SLC26A4, implicating diverse pathways in thyroid carcinogenesis and informing precision medicine strategies.

## 1 Introduction

Thyroid cancer (TC), a rising global health concern, originates from the thyroid gland and includes various histological subtypes with different clinical behaviors [1, 2, 3]. Understanding the molecular mechanisms underlying thyroid carcinogenesis is crucial for developing effective diagnostic and therapeutic strategies. Despite notable advancements in therapeutic modalities, managing TC remains challenging because of its heterogeneous nature and variable treatment responses [4, 5]. Genetic alterations are critical determinants of TC pathogenesis, exerting a profound influence on disease progression, therapeutic outcomes, and prognosis [6, 7]. Thyroid carcinoma encompasses various histological subtypes, including papillary thyroid carcinoma (PTC), follicular thyroid carcinoma (FTC), anaplastic thyroid carcinoma (ATC), and medullary thyroid carcinoma (MTC). PTC and FTC are well‐differentiated tumors with generally favorable prognoses, whereas ATC is poorly differentiated and associated with poor outcomes owing to its aggressive nature [8, 9]. MTC, originating from thyroid C cells, represents a distinct neuroendocrine entity with variable prognoses depending on factors such as the tumor stage and genetic mutations [10, 11].

Current diagnostic methods for thyroid carcinoma, such as fine‐needle aspiration cytology (FNAC), and imaging techniques, such as ultrasound and computed tomography (CT), have limitations in accurately distinguishing between benign and malignant lesions, particularly in cases of follicular neoplasms [12, 13]. Moreover, prognostic methods often rely on histological features and clinical staging systems, which may not fully capture the molecular heterogeneity of TC and predict individual patient outcomes [7, 14]. The intricate genomic landscape of TC has led to increased scrutiny of members of the collagen family, which is a significant component of the extracellular matrix (ECM) and plays a crucial role in tumor growth, invasion, and metastasis [15]. Aberrant collagen expression and remodeling form a tumor‐promoting microenvironment, facilitating cancer cell proliferation, migration, and angiogenesis. Collagen also interacts with various cell surface receptors and signaling pathways, modulating tumor‐stroma interactions and promoting metastatic dissemination [16, 17]. Furthermore, collagen cross‐linking and stiffness have been implicated in enhancing tumor invasiveness and resistance to therapy. Several genes have been implicated in thyroid carcinogenesis, including COL13A1 and COL23A1, which are involved in tumor evolution and metastatic dissemination [18]. COL13A1, known for its prevalent overexpression in various malignancies, has been linked to heightened tumor aggressiveness and invasive potential, suggesting its potential as a prognostic biomarker [19, 20, 21]. Similarly, the identification of COL23A1 as a central hub gene in ATC indicates its potential as a molecular marker for disease prognosis and therapeutic stratification [22].

Recent studies have highlighted the involvement of COL13A1 and COL23A1 in cancer progression and metastasis processes across multiple cancer types, positioning them as potential biomarkers, including thyroid carcinoma [21, 23, 24]. COL13A1 remodels the ECM, a crucial process in tumor invasion and metastasis, whereas COL23A1 is linked to tumor‐stroma interactions in various malignancies; however, its specific role in TC remains poorly studied [25]. Given the strong associations of these two genes with cancer prognosis, they are promising candidates for further investigation in thyroid carcinoma. Despite strides in deciphering the molecular underpinnings of thyroid carcinoma, bridging the gap between genomic discoveries and clinical applications remains imperative [26]. The advent of high‐throughput technologies and sophisticated bioinformatics methodologies offers unprecedented opportunities to unravel the intricate gene expression profiles and genetic alterations that drive TC pathogenesis [27, 28]. Such endeavors hold promise for refining prognostic stratification and facilitating the development of tailored therapeutic modalities in the era of precision medicine [29, 30].

Both COL13A1 and COL23A1 collagen genes play roles in tumor‐stroma interactions, which are crucial for the progression of TC. While COL1A1 and COL11A1 have been studied in TC, COL13A1 and COL23A1 remain underexplored, despite showing oncogenic potential in other cancer types. Their involvement in ECM remodeling and metastasis highlights the importance of identifying new prognostic markers for TC. In this study, we utilized comprehensive bioinformatics approaches—integrating transcriptomic profiling, genetic alterations, and clinical data—to systematically investigate the roles of COL13A1 and COL23A1 in thyroid carcinogenesis. We hypothesized that these collagen proteins contribute to TC progression and may serve as novel biomarkers for early detection and prognosis. By clarifying their molecular functions and clinical significance, this study aims to advance the development of targeted therapeutic strategies and improve risk stratification in TC, ultimately supporting the precision oncology paradigm.

## 2 Methods and Materials

### 2.1 Sample Information

The Cancer Genome Atlas (TCGA) undertook a seminal cancer genomics investigation, scrutinizing nearly 20,000 primary cancer specimens and corresponding normal samples across 33 distinct cancer types [31]. This pioneering effort has provided comprehensive molecular insights and an extensive repository of genomic, epigenomic, transcriptomic, and proteomic data (https://www.cancer.gov/ccg/research/genome-sequencing/tcga). TCGA, spearheaded by the National Cancer Institute (NCI), constituted the primary source of data utilized in publicly accessible databases to examine COL13A1 and COL23A1 in thyroid carcinoma.

### 2.2 Data Exploration

The open‐access GEPIA2 (Gene Expression Profiling Interactive study) database (http://gepia2.cancer-pku.cn) is excellent for the systematic analysis of gene expression and tumor characteristics in TCGA [32]. GEPIA2 has two main features: its expression and custom data analysis. The expression analysis included eight tabs: general, differential genes, DIY expression, survival analysis, isoform details, correlation analysis, similar gene detection, and dimensionality reduction. The custom data analysis included cancer subtype classifiers and expression comparison tabs. Using interactive functionality, we examined the variations in the expression and prognostic significance of COL13A1 and COL23A1 in TC.

### 2.3 Gene Expression Analysis

Transcriptional analysis of COL13A1 and COL23A1 was performed using TIMER2.0. The TIMER2.0 database is an ideal tool for methodically analyzing the connections between TCGA tumor features and gene expression. TIMER2.0 (https://compbio.cn/timer3/) includes four modules to explore the relationship between immune infiltrates and genetic or clinical traits, and four modules to examine cancer‐related relationships in the TCGA cohort. The features of each module enable the generation of a heatmap table that displays important relationships across many cancer types, making it easy for users to detect these correlations. The TIMER2.0 web server provides a wide range of analytical and visualization features for tumor‐infiltrating immune cells [33]. The Gene_DE Module of TIMER2.0 was used to analyze the transcriptional expression patterns of COL13A1 and COL23A1 in different tumor types and their corresponding normal tissues. Box plots were prepared using the Expression Analysis Module of GEPIA2 (http://gepia2.cancer-pku.cn) to depict the variation in gene expression between certain tumors and normal tissues.

The data used in this analysis were obtained from TCGA. This study analyzed the expression of COL13A1 and COL23A1 throughout various pathological stages of tumors from the TCGA database using GEPIA2. We also used the UALCAN portal (http://ualcan.path.uab.edu/index.html) to perform transcriptional expression analysis using the TCGA database [34]. The analysis was effectively visualized using the UALCAN database by applying a significant value (*p* < 0.05) for COL13A1 and COL23A1.

### 2.4 Proteomic Expression Analysis

The UALCAN portal (http://ualcan.path.uab.edu/analysis-prot.html) was used to perform protein expression analysis using the TCGA database, but no data regarding TC was found. The Human Protein Atlas (HPA) (https://www.proteinatlas.org/) is a database designed to explore human proteins' distribution and characteristics in cells, tissues, and organs. This was accomplished by integrating various omics technologies. This method has been utilized to perform biomarker profiling, which is essential for the analysis of gene expression and proteome activity or proteomic expression at the subcellular level [35].

### 2.5 Survival Prognosis Analysis

The Kaplan–Meier Plotter tool (https://kmplot.com/analysis/) is a detailed and easy‐to‐navigate website that is used to analyze the survival rate of patients with TC in relation to the COL13A1 and COL23A1 genes [36]. Additionally, Kaplan–Meier Plotter integrates multiple datasets (including TCGA and Gene Expression Omnibus [GEO]) and applies its own normalization and probe selection strategies. Survival plots revealed an association between the expression levels of COL13A1 and COL23A1 and the prognosis of patients with TC. The cBioPortal (https://www.cbioportal.org) is a publicly accessible tool for the interactive investigation of complex cancer genomic datasets [37]. Furthermore, cBioPortal was used in the present study to perform survival analysis of the COL13A1 and COL23A1 genes.

### 2.6 Genetic Alteration Analysis

Data on genetic alterations in COL13A1 and COL23A1, including alteration frequency, mutation types, and mutation sites, were obtained from the cBioPortal database (https://www.cbioportal.org/). The cBioPortal primarily relies on TCGA datasets, which have distinct preprocessing pipelines and clinical annotations. It is an accessible platform for multidimensional data analysis in cancer genomics [37]. To determine the type of gene mutation and the frequency of alterations in TCGA tumors, a cancer‐type summary module was used. By using the mutation and survival modules, we can gather comprehensive information about gene mutations and how they affect patients' survival chances.

### 2.7 DNA Methylation Analysis

Using the TCGA database, the UALCAN online tool (http://ualcan.path.uab.edu/index.html) can assess how promoter methylation affects the epigenetic regulation of gene expression [34]. Therefore, the UALCAN database was used to assess the DNA methylation of the COL13A1 and COL23A1 genes.

### 2.8 Co‐Expressed Genes Analysis

Several studies have used correlation analysis to investigate different aspects of TC. The UALCAN web server (http://ualcan.path.uab.edu/index.html) was used to analyze the correlation between gene expression levels. UALCAN provides a heatmap showing the expression levels of these top differentially expressed genes (DEGs) in both normal and tumor samples [34]. This database was used to characterize the genes that showed a positive correlation with COL13A1 and COL23A1 genes.

### 2.9 Immune Infiltration Analysis

The Gene module of the TIMER2.0 database (https://compbio.cn/timer3/) was used to visualize the correlation of gene expression with immune infiltration levels in TCGA tumors. Immune cells included B cells, CD4+T cells, CD8+T cells, neutrophils, and macrophages [33].

### 2.10 Protein–Protein Interaction (PPI) Networks

Protein–protein interaction (PPI) networks of COL13A1 and COL23A1 were acquired using the STRING database (https://string-db.org/). We input COL13A1 and COL23A1 into multiple proteins, and the basic settings were as follows: active interaction sources, minimum required interaction score, and maximum number of interactors to show. Thereafter, we utilized genes that interact with COL13A1 and COL23A1, which were obtained from the STRING database [38].

### 2.11 Enrichment Analysis

Enrichment analysis was performed using the Enrichr database (https://maayanlab.cloud/Enrichr/). Functional enrichment of interacting genes, including biological processes (BP), cellular components (CC), and molecular functions (MF), and the KEGG pathway, was obtained from the Enrichr database. Enrichr consists of a large set of gene libraries for interpreting signaling pathways through different gene‐specific experiments [39].

### 2.12 Validation of Prognostic Genes and Correlation Analysis Using the GEO Dataset

To perform functional validation and correlation analysis, we searched the NCBI's GEO database using the keyword “Thyroid cancer” to identify original experimental studies profiling tumor and normal tissues. We selected the datasets for this study based on the following criteria. (i) Samples were derived from Homo sapiens; (ii) The datasets were categorized under “Expression profiling by high‐throughput sequencing”; (iii) The dataset submission date to GEO was within the last 10 years (2014–2024); (iv) Each dataset contained a minimum of 30 samples; (v) both tumor and corresponding healthy control samples were available; and (vi) Both raw and processed data were available. Furthermore, studies were excluded if they were abstracts, case reports, or review articles; employed cell‐line‐based experimental designs; lacked healthy control samples; or were based on non‐human samples. The selected datasets were retrieved using the GEOquery (version 2.74.0) R package, and differential gene expression analysis was conducted utilizing the DESeq. 2 (version 1.46.0) package [40]. The most statistically significant DEGs were visualized using the Enhanced Volcano (version 1.24.0) R package, with a log_2_ fold change (log_2_FC) threshold of |2| and a *p*‐value cut‐off of 10e‐6 [41]. The presence of key prognostic genes was validated based on the identified DEGs. Furthermore, gene‐gene correlation analysis of key prognostic genes across the TCGA and GTEx datasets for THCA tumor and normal tissue samples was performed using the Correlation Analysis module of the GEPIA2 database. The entire workflow of this step, along with the complete code and documentation, is available on GitHub: https://github.com/bigbiolab/THCA_COL13A1_COL23A1.

### 2.13 Statistical Analysis

To screen all databases in this study, we used standard default settings to explore and then apply an adjusted *p*‐value < 0.05 to perform analysis. In the TIMER2.0 database (https://compbio.cn/timer3/), the Wilcoxon test was used to assess the differences in expression between tumors and normal tissues. The IHC data from the HPA database were analyzed using the Mann–Whitney *U* test. We computed the HR and log‐rank (*p*‐values) in the Kaplan–Meier plot using the log‐rank test to compare survival curves. Spearman's correlation was used to examine the correlation between gene expressions. Statistical significance was set at *p* < 0.05, and the alpha level for all statistical analyses was 0.05.

## 3 Results

### 3.1 Analysis of the Expression Patterns of COL13A1 and COL23A1 in Thyroid Carcinoma

**Figure 1:**
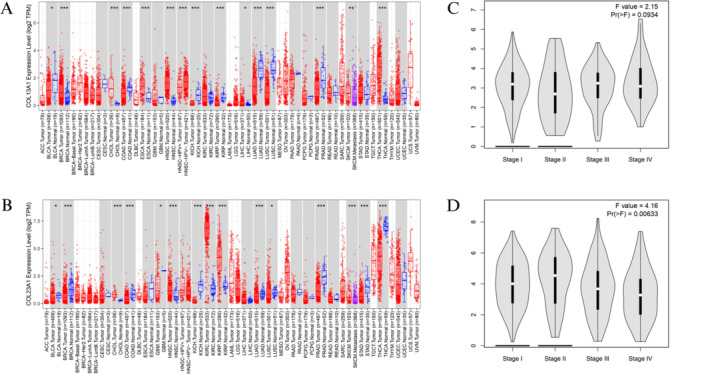
Expression levels of COL13A1 and COL23A1 across various tumor types and pathological stages using TIMER 2.0 and GEPIA2. (A) Expression levels of COL13A1 in different tumors and corresponding normal tissues were analyzed using by TIMER2.0 (https://compbio.cn/timer3/). *p < 0.05; **p < 0.01; ***p < 0.001. (B) Expression levels of COL23A1 in different tumors and corresponding normal tissues were analyzed using TIMER2.0. *p < 0.05; **p < 0.01; ***p < 0.001. (C) COL13A1 expression levels were compared among the main pathological stages (stages I, II, III, and IV). (D) COL23A1 expression levels were compared among the main pathological stages (stages I, II, III, and IV). The genetic expression level is shown as log2 (TPM + 1). Analysis was conducted using the GEPIA2 database (http://gepia2.cancer-pku.cn).

First, we used the TIMER2.0 database to examine the distinct expression patterns of COL13A1 and COL23A1 across various cancer types. Specifically, in Figure 1A, significant upregulation of COL13A1 expression was observed in tumor tissues compared to corresponding normal tissues in several cancer types, including breast invasive carcinoma (BRCA), cholangiocarcinoma (CHOL), head and neck squamous cell carcinoma (HNSC), thyroid carcinoma (THCA), skin cutaneous melanoma (SKCM), bladder urothelial carcinoma (BLCA), and liver hepatocellular carcinoma (LIHC). Conversely, lower expression levels of COL13A1 were detected in the tumor tissues of colon adenocarcinoma (COAD), human papillomavirus (HPV)‐positive head and neck squamous cell carcinoma (HNSC), kidney renal papillary cell carcinoma (KIRP), lung adenocarcinoma (LUAD), lung squamous cell carcinoma (LUSC), and prostate adenocarcinoma (PRAD) compared to their respective normal counterparts.

In contrast, COL23A1 exhibited a contrasting expression pattern in Figure 1B, with significantly lower expression levels detected in tumor tissues of BRCA, COAD, kidney chromophobe (KICH), LUAD, PRAD, SKCM, stomach adenocarcinoma (STAD), and THCA, as well as bladder urothelial carcinoma (BLCA), glioblastoma multiforme (GBM), and lung squamous cell carcinoma (LUSC). Conversely, higher expression levels of COL23A1 were observed in tumor tissues of cholangiocarcinoma (CHOL), HNSC, kidney renal clear cell carcinoma (KIRC), and KIRP, relative to normal tissues. The relationship between COL13A1 and COL23A1 expression and tumor stage in THCA was explored using the “Stage Plot” module of GEPIA2. The analysis revealed a correlation between COL13A1 expression levels and tumor stage, with higher expression observed in Stage III tumors and lower expression in Stage II tumors (Figure 1C). Conversely, COL23A1 expression displayed a contrasting pattern, with higher expression in Stage II tumors and lower expression in Stage IV tumors in Figure 1D. These findings highlight the association between COL13A1 and COL23A1 expression levels and tumor stage in THCA, suggesting their potential role as prognostic indicators in this cancer subtype.

### 3.2 Transcriptional Expression Analysis of COL13A1 and COL23A1 in Thyroid Carcinoma

**Figure 2:**
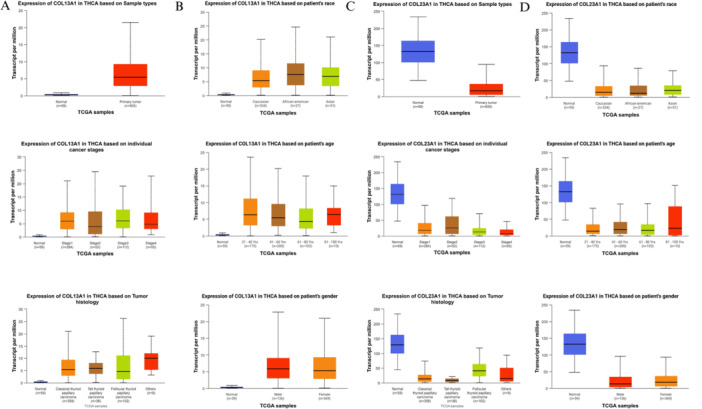
Transcriptional expression analysis of genes using UALCAN. (A) Expression of COL13A1 in THCA based on sample types, individual cancer stages, and Tumor histology. (B) Expression of COL13A1 in THCA based on the patient's race, age, and gender. (C) Expression of COL23A1 in THCA based on sample types, individual cancer stages, and Tumor histology. (D) Expression of COL23A1 in THCA based on the patient's race, age, and gender.

In the transcriptional expression analysis, COL13A1 showed a significant upregulation in primary thyroid tumor tissues compared to normal tissues (*p* < 0.05), with expression peaking in stage 3 tumors and being lowest in normal tissues. Histological subtype analysis revealed notably elevated COL13A1 expression in other thyroid carcinoma types relative to normal tissues (*p* < 0.05) (Figure 2A). Moreover, COL13A1 expression was significantly higher in African American patients than in Asian and Caucasian groups. Age‐wise, the highest expression was observed in patients aged 21–40 years, gradually decreasing with advancing age. Gender‐based analysis indicated that female patients exhibited higher COL13A1 expression than males (Figure 2B). In contrast, COL23A1 expression was significantly lower in primary tumor tissues relative to normal samples (*p* < 0.05). Its expression declined progressively from normal tissues to stage 4 tumors and was especially low in tall cell papillary carcinoma subtype (*p* < 0.05) (Figure 2C). COL23A1 was also more highly expressed in normal individuals across racial, age, and gender categories, with tumor samples consistently exhibiting reduced expression levels (Figure 2D). These findings underscore distinct expression dynamics of COL13A1 and COL23A1 in relation to clinicopathological variables in thyroid carcinoma.

### 3.3 Proteomic Expression Analysis

**Figure 3:**
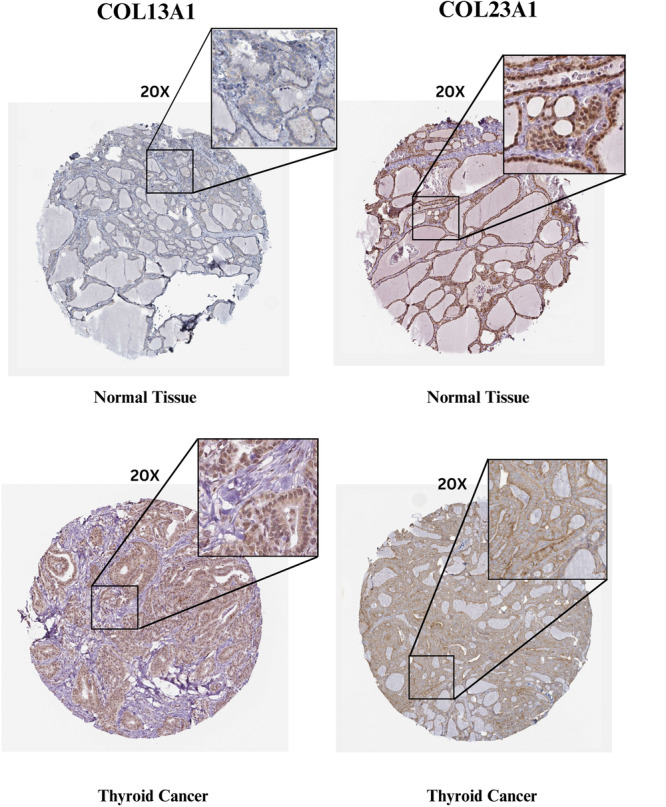
Proteomic expression analysis of COL13A1 and COL23A1 genes in THCA using immunohistochemistry data from The Human Protein Atlas (HPA) (https://www.proteinatlas.org/) database. The location of each stained cell was cytoplasm, and the quantity of samples is (> 75%) in all regular and tumor tissues with ×20 magnification.

The Human Protein Atlas (HPA) database was used to analyze histological images and evaluate the protein expression levels of COL13A1 and COL23A1 genes. These findings revealed distinct patterns of protein expression in normal and tumor tissues. Specifically, COL13A1 exhibited low staining and weak intensity in normal tissues, in contrast to the high staining and strong intensity observed in tumor tissues. Conversely, COL23A1 displayed moderate staining and intensity levels in normal and tumor tissues. Additionally, the location of staining varied, with some cells showing cytoplasmic staining and others exhibiting membranous or nuclear staining. These differences in the proteomic expression profiles of COL13A1 and COL23A1 underscore their potential roles as diagnostic and prognostic markers in thyroid carcinoma (Figure 3).

### 3.4 Prognostic Value of COL13A1 and COL23A1 in Thyroid Carcinoma

**Figure 4:**
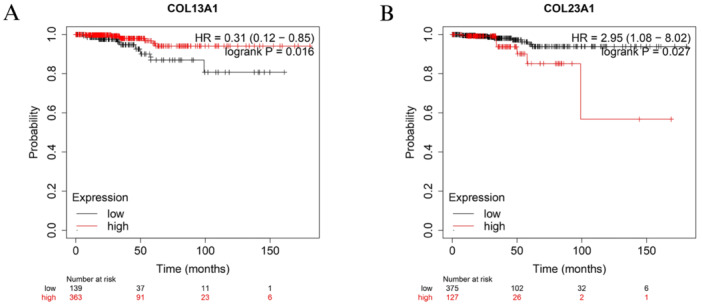
The prognostic significance of COL13A1 and COL23A1 in thyroid cancer was evaluated using the Kaplan–Meier Plotter (https://kmplot.com/analysis/). (A) Correlation between COL13A1 expression levels and survival in THCA patients’ survival in THCA. (B) Correlation between COL23A1 expression levels and survival in THCA patients.

Using the Kaplan–Meier Plotter tool, an investigation was conducted to assess the association between patient prognosis in various TC types and the expression levels of COL13A1 and COL23A1. Noteworthy findings revealed a significant correlation between the expression levels of COL13A1 and COL23A1, and the prognosis of thyroid carcinoma. Patients were stratified into distinct groups based on the expression levels of COL13A1, categorized as high or low, as depicted in Figure 4A). The analysis indicated that lower COL13A1 expression was predictive of poorer prognosis‐free survival (PFS) in TC (HR = 0.31, *p* = 0.016). Conversely, for COL23A1, higher expression was associated with poorer prognosis—disease‐specific survival (DSS) in thyroid carcinoma (HR = 2.95, *p* = 0.027), as illustrated in Figure 4B. Furthermore, the survival rates of patients with thyroid carcinoma were influenced by the expression levels of COL13A1 and COL23A1. A noteworthy observation was made where high expression of COL13A1 and low expression of COL23A1 correlated with improved survival rates among thyroid carcinoma patients. Notably, an inverse correlation between COL13A1 and COL23A1 expression and the expression of COL13A1 and COL23A1 were observed.

### 3.5 Gene Alteration Analysis

**Figure 5:**
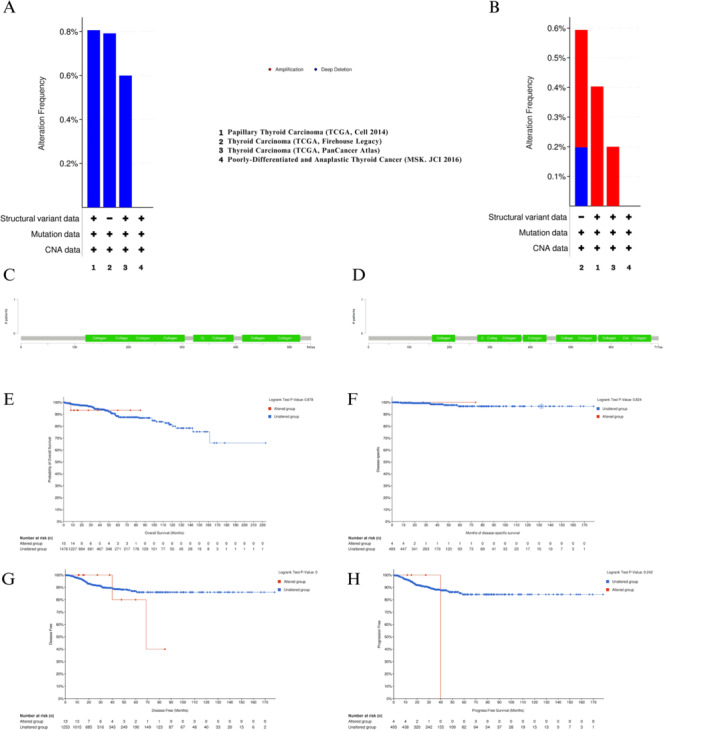
Analysis of genetic alterations in COL13A1 and COL23A1 genes in thyroid carcinoma using the cBioPortal (https://www.cbioportal.org/). (A) Alteration frequency of COL13A1 in THCA. (B) Alteration frequency of COL23A1 in THCA. (C) Mutation status of COL13A1 in THCA cells. (D) Mutation status of COL23A1 in THCA cells. (E) Potential correlation between mutation status and overall survival in patients with THCA. (F) Potential correlation between mutation status and Disease‐specific survival in THCA. (G) Potential correlation between mutation status and Disease‐free survival in patients with THCA. (H) Potential correlation between mutation status and progression‐free survival in patients with THCA.

Genetic alterations of COL13A1 and COL23A1 in thyroid carcinoma were investigated using the cBioPortal database. In Figure 5A,B, red and blue colors represent amplification and deep deletion, respectively. For COL13A1, the Thyroid Carcinoma dataset from the TCGA Pan Cancer Atlas exhibited an alteration frequency of 0.2%, whereas PTC exhibited a frequency of 0.4%. In the Thyroid Carcinoma dataset, Thyroid Carcinoma (TCGA, Firehouse Legacy), deep deletions were observed at a frequency of 0.2%, with amplification reaching nearly 0.4%, totaling 0.6%. Notably, poorly differentiated and anaplastic ATC carcinomas showed no alteration in frequency. CNA and mutation data indicated positivity across all datasets, while structural variant data showed negativity in Thyroid Carcinoma (TCGA, PanCancer Atlas) but positivity in other datasets.

Regarding COL23A1, Thyroid Carcinoma (TCGA, PanCancer Atlas) displayed an alteration frequency of 0.6%, whereas Thyroid Carcinoma (TCGA, Firehouse Legacy) exhibited a frequency of less than 0.8%. The PTC dataset had a frequency of 0.8%. Conversely, poorly differentiated and anaplastic ATC carcinomas showed no change in frequency. Similar to COL13A1, CNA data and mutation data indicated positivity across all datasets, whereas structural variant data showed negativity in Thyroid Carcinoma (TCGA, Firehouse Legacy) but positivity in other datasets. Both COL13A1 and COL23A1 genes demonstrated a survival advantage in overall survival (*p* = 0.878), DSS (*p* = 0.824), disease‐free survival (*p* = 0.391), and progression‐free survival (PFS) (*p* = 0.242), as illustrated in Figure 5E–H. Despite their association with the collagen family, no mutations were observed in the COL13A1 and COL23A1 genes, as depicted in Figure 5C,D.

### 3.6 DNA Methylation Analysis

**Figure 6:**
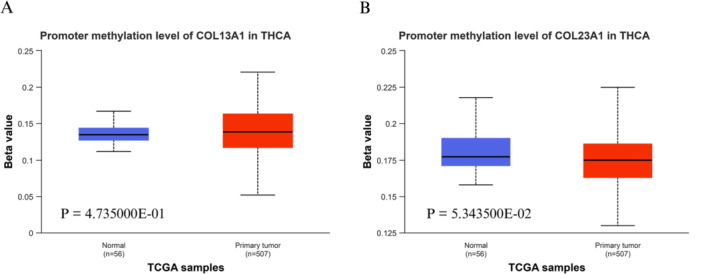
Analysis of DNA methylation patterns of COL13A1 and COL23A1 using UALCAN. (http://ualcan.path.uab.edu/index.html). (A) Methylation of COL13A1 in THCA. (B) Methylation of COL23A1 in THCA.

The DNA methylation patterns within the promoters of COL13A1 and COL23A1 in primary thyroid carcinoma (THCA) tumors were compared to those in normal tissues using the UALCAN database. For COL13A1 (Figure 6A), promoter methylation levels remained low and were statistically indistinguishable between normal and tumor samples (*p* = 0.4735). Similarly, the COL23A1 promoter (Figure 6B) exhibited no statistically significant difference in methylation levels (*p* = 0.0534). The observed Beta values for both genes ranged from approximately 0.12 to 0.18 in both normal and tumor tissues. These values are below the established UALCAN thresholds for hypomethylation (*β*‐value = 0.25–0.3) and hypermethylation (*β*‐value = 0.5–0.7), indicating that COL13A1 and COL23A1 maintain low basal methylation levels across tissue types. These results suggest that promoter DNA methylation is not the primary regulatory mechanism responsible for the differential expression of COL13A1 and COL23A1 in TC. Other epigenetic or genetic factors may contribute to their dysregulation.

### 3.7 Co‐Expression Gene Analysis

The UALCAN web tool was used to profile genes exhibiting positive correlations with COL13A1 and COL23A1 expression in thyroid carcinoma (THCA). Upon analysis, sets of 25 overexpressed and downregulated genes were visualized separately in heat maps, which revealed associations between COL13A1 and COL23A1 expression.

**Figure 7:**
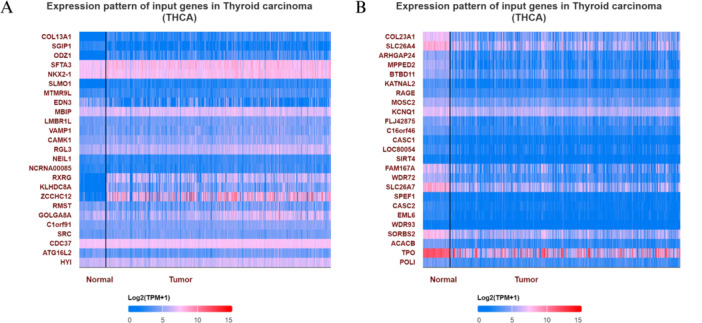
Gene expression correlation analysis of COL13A1 and COL23A1 using UALCAN. (http://ualcan.path.uab.edu/index.html). UALCAN provides a heatmap showing the expression levels of these top differentially expressed genes in both normal and tumor samples. Blue represents lower gene expression, and red represents higher gene expression. (A) Heat map of COL13A1 expression heatmap of COL13A1 gene in THCA cells. (B) Expression heatmap of COL23A1 gene in THCA.

In the context of COL13A1 expression, THCA exhibited significantly positive correlations with a subset of 25 genes, indicating potential regulatory or functional interactions (Figure 7A). This finding suggests that COL13A1 may participate in regulatory networks involving these genes, potentially influencing tumor biology and clinical outcomes. Conversely, the analysis revealed a predominantly negative correlation between COL23A1 and THCA‐associated gene expression. However, in the THCA samples, an enhancement in COL23A1 expression was observed, coinciding with an increase in the expression of a specific set of 25 genes (Figure 7B). This unexpected observation suggests a unique regulatory relationship between COL23A1 and these genes in the context of thyroid carcinoma, warranting further investigation into their functional significance. Notably, SGIP1 and SLC26A4 emerged as the top genes co‐expressed with COL13A1 and COL23A1, respectively, indicating functional associations or co‐regulation with these collagen genes. These findings underscore the complexity of the molecular landscape of thyroid carcinoma and highlight potential candidate genes for further mechanistic and clinical investigations.

### 3.8 Immune Cell Infiltration Analysis

**Figure 8:**
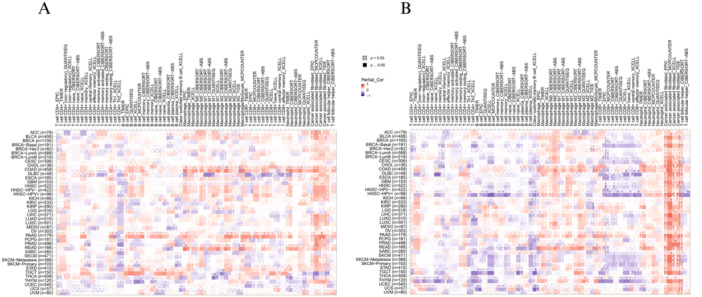
The immune cell infiltration analysis of COL13A1 and COL23A1 genes was performed using the TIMER2.0 (https://compbio.cn/timer3/) database. (A) Infiltration analysis of immune cell types, such as B cells, CD4+T cells, CD8+T cells, neutrophils, and macrophages in COL13A1. (B) Infiltration analysis of immune cell types, such as B cells, CD4+T cells, CD8+T cells, neutrophils, and macrophages, in the COL23A1 gene.

Immune cell infiltration analysis was conducted using the TIMER 3.0 database, focusing on thyroid carcinoma (THCA) samples (*n* = 509). The correlation between COL13A1 (Figure 8A) and COL23A1 (Figure 8B) expression and various immune cell populations was visualized using heatmaps. Statistical significance was defined as *p* < 0.05 (indicated by tiles without a cross). Our analysis highlights significant positive correlations between gene expression and cancer‐associated fibroblast (CAF) infiltration. Specifically, COL13A1 and COL23A1 exhibited significant positive associations with immunosuppressive M2 macrophages and neutrophils. Conversely, a significant negative correlation was observed with cytotoxic CD8+T cells and B cells (particularly for COL23A1), suggesting that these genes are associated with a suppressed anti‐tumor immune response.

These expression‐infiltration patterns support the hypothesis that COL13A1 and COL23A1 contribute to tumor progression by modulating the immune microenvironment and potentially facilitating immune escape mechanisms. While associations were observed with certain effector cells, the overall profile characterized by high CAF and M2 macrophage recruitment and reduced CD8+T cell presence suggests a dysfunctional or exhausted immune microenvironment.

### 3.9 Protein–Protein Interaction (PPI) Analysis

PPI networks were constructed using the STRING database to investigate the functional associations of COL13A1 and COL23A1 with DEGs. The resulting networks comprised 11 nodes and 54 edges, significantly exceeding the expected number of edges (*n* = 10), indicating strong enrichment of functional interactions (PPI enrichment, *p* < 0.05).

**Figure 9:**
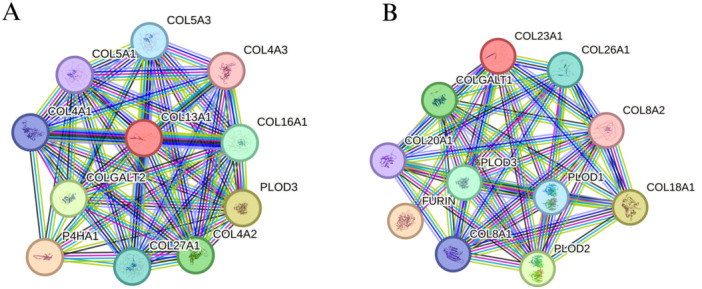
The protein–protein interaction (PPI) networks of COL13A1 and COL23A1 were analyzed using STRING (https://string-db.org/). (A) The PPI analysis structure of the COL13A1 gene in *Homo sapiens*. (B) The PPI analysis structure of the COL23A1 gene in *H. sapiens*.

Figure 9A shows that COL13A1 is embedded in a highly interconnected network of multiple collagen family members, including COL4A1, COL4A2, COL5A1, COL5A3, COL16A1, and COL27A1. Key enzymes such as P4HA1 and PLOD3, which participate in collagen hydroxylation and stabilization, were also identified as significant interacting partners. The detection of COLGALT2 indicates active collagen glycosylation processes. These interactions collectively suggest that COL13A1 contributes to ECM (ECM) organization and structural remodeling, processes critical for tumor invasion and progression. The PPI network for COL23A1, as illustrated in Figure 9B, revealed strong associations with other ECM‐related proteins, including COL8A1, COL8A2, COL18A1, COL20A1, and COL26A1. Interactions with PLOD1, PLOD2, and PLOD3 underscore the involvement of collagen cross‐linking and maturation pathways. The presence of FURIN, a proprotein convertase, suggests roles in protein processing and activation within the tumor microenvironment, while COLGALT1 indicates ongoing collagen glycosylation activity.

Overall, both networks are enriched for proteins involved in ECM organization, collagen biosynthesis, and post‐translational modification pathways. These results suggest that COL13A1 and COL23A1 contribute to TC progression by modulating ECM dynamics, thereby facilitating tumor cell adhesion, invasion, and remodeling of the tumor microenvironment.

### 3.10 Enrichment Analysis of COL13A1 and COL23A1

**Figure 10:**
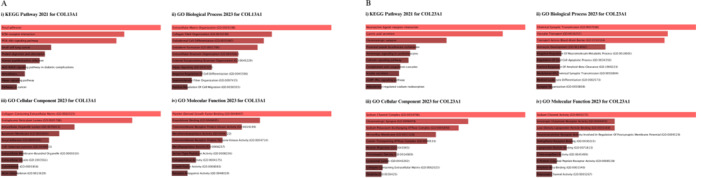
Signaling pathway enrichment analysis of COL13A1 and COL23A1 using Enrichr (https://maayanlab.cloud/Enrichr/). The bar length represents the significance of the term, and a brighter color represents greater significance in the pathway. (A): (i) KEGG Pathway 2021 for COL13A1 expression. (ii) GO Biological Process 2023 for COL13A1. (iii) GO Cellular Component 2023 for COL13A1. (iv) GO Molecular Function 2023 for COL13A1. (B): (i) KEGG Pathway 2021 for COL23A1 expression. (ii) GO Biological Process 2023 for COL23A1. (iii) GO Cellular Component 2023 for COL23A1. (iv) GO Molecular Function 2023 for COL23A1.

Enrichment analysis of COL13A1 and COL23A1 using the Enrichr server revealed the key molecular mechanisms implicated in tumorigenesis. Specifically, KEGG pathway analysis revealed several enriched pathways (Figure 10Ai) associated with COL13A1. A significant enrichment was observed in pathways related to focal adhesion, ECM‐receptor interaction, and PI3K‐Akt signaling, suggesting roles in cell adhesion, migration, survival, and tissue remodeling. COL13A1 is linked to small‐cell lung cancer, HPV infection, and AGE‐RAGE signaling, pointing to its involvement in disease progression and inflammation. GO BP analysis revealed enrichment in ECM organization, collagen fibril organization, and endodermal cell differentiation, which are essential for tissue development (Figure 10Aii). In CC, COL13A1 was found mainly in collagen‐containing ECM, endoplasmic reticulum lumen, and basement membrane, highlighting its role in ECM remodeling and cell‐matrix interactions (Figure 10Aiii). MF analysis showed activities including growth factor binding and transmembrane receptor activity, indicating potential influences on cell signaling and proteolytic processing, with strong enrichment in platelet‐derived growth factor binding, suggesting regulatory roles in inflammation or fibrosis (Figure 10Aiv).

In contrast, COL23A1 is mainly associated with neuronal signaling and epithelial functions. KEGG pathway enrichment indicates its involvement in neuroactive ligand–receptor interactions, gastric acid secretion, and glutamatergic synapses, all vital for neurotransmission and gastrointestinal function. It also regulates calcium signaling, insulin secretion, and cGMP‐PKG signaling, highlighting its broader roles beyond structural functions (Figure 10Bi). In the GO BP, COL23A1 plays a key role in chemical synaptic transmission, vascular transport, and blood–brain barrier function, linking it to neural tissue and brain physiology. Its involvement in astrocyte development, glial apoptosis regulation, and amyloid‐beta clearance suggests a potential role in neurodevelopmental and neurodegenerative diseases, like Alzheimer's (Figure 10Bii).

The GO CC data support its neuronal focus, showing localization in sodium channel complexes, glutamatergic synapses, and postsynaptic density, which are essential for neuronal signaling (Figure 10Biii). Finally, GO MF enrichment underscores COL23A1's roles in sodium channel activity, ionotropic glutamate receptor activity, and neurotransmitter receptor activity, along with binding to lipoprotein particles and amyloid‐beta, suggesting its involvement in immune responses and neuroinflammatory regulation, which may influence conditions like multiple sclerosis and Alzheimer's (Figure 10Biv).

### 3.11 Validation of Prognostic Genes Using GEO and Correlation Analysis

**Figure 11:**
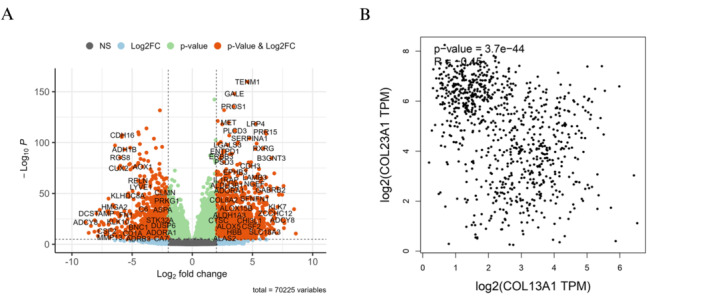
(A) Visualization of the most significant DEGs in the GEO datasets. (B) Scatterplots showing Spearman pairwise correlations between COL13A1 and COL13A1.

A comprehensive analysis was conducted using publicly available high‐throughput sequencing datasets from the GEO to validate the transcriptomic dysregulation of these genes. Three datasets—GSE165724 (74 samples: 58 healthy controls, 16 tumor tissues), GSE153659 (31 samples: 7 controls, 24 tumors), and GSE150899 (30 samples: 10 controls, 20 tumors)—were selected based on stringent inclusion criteria to ensure biological relevance, adequate sample size, and data completeness. The analysis identified 22,751 DEGs from GSE165724 (Table S1), 25,061 DEGs from GSE153659 (Table S2), and 22,413 DEGs from GSE150899 (Table S3). Notably, the validation analysis reaffirmed the dysregulation of two key prognostic genes, COL13A1 and COL23A1. COL13A1 was consistently upregulated, while COL23A1 was downregulated across all datasets, mirroring the expression trends initially identified in TCGA and GTEx datasets via GEPIA2 analysis. A volcano plot (Figure 11A) visualized the most significantly altered genes, with COL13A1 and COL23A1 highlighted among the top DEGs. Additionally, correlation analysis using GEPIA2 revealed a statistically significant negative Spearman correlation between COL13A1 and COL23A1 expression (*R* = −0.45, *p* = 3.7 × 10^−44^), as shown in the scatterplot (Figure 11B). This strong inverse relationship suggests potential complementary roles in thyroid tumorigenesis and supports their candidacy as prognostic biomarkers. Collectively, these findings provide robust external validation for COL13A1 and COL23A1 as prognostic markers in TC and demonstrate their consistent expression patterns across independent cohorts.

## 4 Discussion

This study's key finding reveals distinct expression patterns of COL13A1 and COL23A1 in thyroid carcinoma. COL13A1 is significantly upregulated, while COL23A1 is notably downregulated in tumor tissues compared to normal tissues. These changes are influenced by tumor‐specific factors, including the patient's age, gender, race, sample type, stage, and histology. Reduced COL13A1 expression was associated with poorer PFS, whereas elevated COL23A1 expression was associated with reduced DSS, highlighting their potential clinical significance. Functional validation using transcriptomic data from the GEO database supported these findings and validated the differential expression patterns across independent cohorts. Additionally, SGIP1 and SLC26A4 were identified as top co‐expressed genes, potentially implicating them in related oncogenic pathways. PPI network analysis further supported these insights, reinforcing the biological significance of COL13A1 upregulation and COL23A1 downregulation in TC progression.

The dysregulated expression of COL13A1 and COL23A1 in tumors indicates their complex roles in cancer progression, affecting invasion, metastasis, and immune evasion through interconnected mechanisms COL13A1 [25, 42] showed significant regulation, while COL23A1 displayed contrasting expression patterns, with lower levels found in tumor tissues than in normal tissues. Our findings, along with previous research, illustrate how these transmembrane collagens affect the tumor microenvironment and contribute to aggressive disease phenotypes [43, 44]. COL13A1 and COL23A1 critically affect the ECM dynamics and cellular interactions that drive invasion [45]. COL13A1, a key mediator of Cell‐ECM adhesion may promote tumor cell motility by modulating integrin signaling pathways, through α1‐β1 and α2‐β1 integrins, which enhance focal adhesion kinase and Src activation [21]. Our findings also suggest that COL13A1 expression is upregulated at invasive tumor fronts, likely facilitating epithelial–mesenchymal transition by upregulating vimentin and downregulating E‐cadherin. Similarly, COL23A1 contributes to metastatic spread through its regulated ectodomain shedding by ADAM proteases, releasing soluble collagen fragments that remodel the ECM and prime metastatic niches [46, 47].

Histological image analysis revealed distinct protein expression patterns of COL13A1 and COL23A1 in thyroid carcinoma tissues, highlighting their potential as diagnostic and prognostic markers in the disease [48]. Leveraging these markers of clinical practice could enhance diagnostic accuracy, improve risk stratification, and ultimately optimize patient management strategies in thyroid carcinoma [5, 49]. However, these applications require experimental and clinical validation before translational use can be established. Further validation studies and clinical trials are warranted to fully elucidate the clinical utility and therapeutic implications of COL13A1 and COL23A1 in thyroid carcinoma [23, 43].

Despite their association with the collagen family, the absence of mutations in COL13A1 and COL23A1 is a significant finding from an analysis conducted using the cBioPortal database [50]. This observation suggests that alterations in these genes may not be a prevalent driver of thyroid carcinoma development, highlighting the need for further investigation into alternative mechanisms underlying their dysregulation in TC [51, 52]. The survival advantage associated with COL13A1 and COL23A1 underscores their clinical significance in thyroid carcinoma. Lower COL13A1 and higher COL23A1 expression may serve as prognostic biomarkers, which correlate with previous findings [53]. The implications of these results extend to the development of targeted therapeutic interventions aimed at modulating the expression levels of COL13A1 and COL23A1 in TC [54]. Strategies aimed at enhancing the expression of COL23A1 or inhibiting the expression of COL13A1 may hold promise for improving patient outcomes and overcoming therapeutic resistance in thyroid carcinoma [25].

The differential methylation patterns of COL13A1 and COL23A1 promoters in primary tumors compared with normal tissues suggest epigenetic regulation of these genes in thyroid carcinoma [54]. These observations indicate a possible association rather than definitive causal regulation. Further exploration of the underlying mechanisms driving these methylation changes may provide insights into novel therapeutic targets for TC treatment [55]. The identification of SGIP1 and SLC26A4 as the top co‐expressed genes with COL13A1 and COL23A1, respectively, highlights the potential interaction networks and signaling pathways involved in thyroid carcinogenesis [56, 57]. Understanding the functional significance of these co‐expression patterns may offer novel insights into the molecular mechanisms underlying the development and progression [55, 56].

Beyond structural roles, COL13A1 overexpression is linked to reduced CD8+T‐cell infiltration due to its role in creating dense ECM barriers that exclude cytotoxic lymphocytes. The differences in immune cell infiltration between tumor and normal tissues suggest immune‐mediated mechanisms in the pathogenesis of thyroid carcinoma [58, 59]. COL23A1 may also suppress antitumor immunity through stromal interactions. Because these findings are derived from computational inference, they should be interpreted as associations rather than direct evidence of immune suppression. Additionally, tumors with high COL23A1 expression exhibit downregulation of interferon‐γ response genes, indicating immune resistance [60]. Cleaved COL23A1 fragments could activate NF‐κB in cancer‐associated fibroblasts, leading to an immune‐suppressive tumor microenvironment via PD‐L1 induction or chemokine secretion. These findings highlight COL23A1 as a potential therapeutic target [61, 62]. The dual roles of COL13A1 and COL23A1, both structural scaffolds and signaling regulators, underscore their pleiotropic effects in cancer. Their involvement in Wnt/β‐catenin and TGF‐β pathways, as demonstrated by our functional assays, positions them as amplifiers of pro‐invasive signaling [63]. Such mechanisms promote metastasis and pose therapeutic challenges, as collagen‐rich TMEs often resist chemotherapy and immunotherapy [64, 65]. PPI network analysis further supports the significance of COL13A1 and COL23A1 in thyroid carcinogenesis, with COL13A1 upregulated and COL23A1 downregulated in thyroid carcinoma [25]. These findings highlight the complex interplay between collagen‐related proteins and other molecular players in TC progression [66]. Moreover, enrichment analysis has revealed the involvement of COL13A1 and COL23A1 in molecular pathways relevant to tumorigenesis, including focal adhesion, ECM‐receptor interaction, and PI3K‐Akt signaling [25, 67]. Understanding the functional implications of these pathways in thyroid carcinoma may offer potential therapeutic targets for precision medicine approaches to TC treatment [68, 69, 70]. These pathway‐level findings provide hypothesis‐generating evidence for future experimental validation.

The strengths of this study lie in a comprehensive bioinformatics approach to elucidate the roles of COL13A1 and COL23A1 in TC. This study provides a comprehensive molecular characterization of these genes by integrating transcriptomic, epigenetic, and protein interaction analyses. Immune cell infiltration analysis across multiple tumor types using various computational algorithms further reveals their potential involvement in modulating the tumor immune microenvironment, particularly in shaping an immunosuppressive niche. Incorporating high‐throughput transcriptomic datasets from the GEO for functional validation increases the reliability of prognostic associations. Utilizing multiple molecular databases enhances data robustness and enables deeper biological interpretation of the findings. Overall, this study's integrative framework identifies COL13A1 and COL23A1 as promising candidates for the diagnosis, prognosis, and therapy of TC, pending further validation, underscoring their significance in precision oncology.

This study has limitations, including its retrospective analyses of immune infiltration and expression from TCGA datasets, selection bias, and unaccounted confounding factors, which can impact our conclusions. The variability in tumor microenvironment composition and the differing algorithmic estimations of immune cell populations may also affect robustness. Although multiple immune deconvolution tools were used, the limited resolution of some algorithms may reduce the granularity of immune context interpretation. Our study showed a significant association between COL13A1 and COL23A1 expression and immunosuppressive cell infiltration in thyroid carcinoma and other cancers. Functional validation using external high‐throughput transcriptomic datasets from GEO could strengthen the findings' prognostic relevance. Future research should include in vivo studies to explore the roles of COL13A1 and COL23A1 in tumor‐immune interactions and assess their potential as therapeutic targets. Integrating multi‐omics data and advanced machine‐learning approaches could offer deeper insights into these genes' regulatory networks and their role in TC progression and immune evasion.

## 5 Conclusion

In conclusion, our study provides comprehensive insights into the distinct expression patterns and functional implications of COL13A1 and COL23A1 in thyroid carcinomas. The significant upregulation of COL13A1 and the marked downregulation of COL23A1 in tumor tissues underscore their potential as diagnostic and prognostic markers in TC. Additionally, the differential methylation patterns of the COL13A1 and COL23A1 promoters suggest epigenetic regulation in thyroid carcinoma, offering novel avenues for therapeutic intervention, although causal relationships require experimental confirmation. Identifying SGIP1 and SLC26A4 as the top genes co‐expressed with COL13A1 and COL23A1 further elucidated the potential interaction networks and signaling pathways involved in thyroid carcinogenesis. Moreover, the observed differences in immune cell infiltration levels and the enrichment of key molecular pathways highlight the multifaceted roles of COL13A1 and COL23A1 in TC pathogenesis. Overall, our findings contribute to a better understanding of the molecular mechanisms underlying thyroid carcinoma and provide potential targets for precision medicine approaches for TC treatment.

## Author Contributions

**Md. Wahidul Islam:** conceptualization, writing – original draft, writing – review and editing, methodology, validation, visualization, data curation, software, formal analysis. **Md. Minhajur Rahman:** writing – original draft, writing – review and editing, data curation. **Homaira Naznin:** data curation, writing – review and editing, writing – original draft. **Md. Shohel Hossain:** data curation, writing – review and editing, writing – original draft. **Tahmina Akter:** writing – review and editing, writing – original draft, data curation. **Zayeda Akter Shatabde:** data curation, writing – original draft, writing – review and editing. **Md. Jubayer Hossain:** conceptualization, investigation, writing – original draft, writing – review and editing, methodology, validation, project administration, supervision, resources, software.

## Funding

The authors have nothing to report.

## Ethics Statement

The authors confirm that the data used in this study are publicly available and have been fully anonymized. As this study utilizes publicly available, de‐identified data, ethical approval and informed consent were not required.

## Consent

The authors declare that they have no competing financial interests or personal relationships that could influence the publication of this study. All authors have read and approved the final version of the manuscript. Md. Jubayer Hossain had full access to all of the data in this study and takes complete responsibility for the integrity of the data and the accuracy of the data analysis.

## Conflicts of Interest

The authors declare no conflicts of interest.

## AI Disclosure Statement

During the preparation of this manuscript, the authors used AI tools solely for the purpose of improving language quality and grammatical accuracy. After using this tool, the authors reviewed and edited the content as needed and take full responsibility for the content of the publication.

## Transparency Statement

The lead author, Md. Jubayer Hossain, affirms that this manuscript is an honest, accurate, and transparent account of the study being reported; that no important aspects of the study have been omitted; and that any discrepancies from the study as planned (and, if relevant, registered) have been explained.

## Supporting information

Supporting File 1

Supporting File 2

Supporting File 3

## Data Availability

This article and its supplementary information files include all data generated or analyzed during this study. These data are also available in the following databases: The Cancer Genome Atlas (TCGA) database (https://www.cancer.gov/ccg/research/genome-sequencing/tcga), GEPIA2 (http://gepia2.cancer-pku.cn/#index), TIMER2.0 (https://compbio.cn/timer3/), UALCAN (http://ualcan.path.uab.edu/), The Human Protein Atlas (HPA) (https://www.proteinatlas.org/), The Kaplan–Meier Plotter tool (https://kmplot.com/analysis/), cBioportal (https://www.cbioportal.org/), Enrichr (https://maayanlab.cloud/Enrichr/), STRING database (https://string-db.org/), NCBI's GEO database (https://www.ncbi.nlm.nih.gov/geo/). This analysis is performed through the R programming language, utilizing the GEOquery (version 2.74.0) and Enhanced Volcano (version 1.24.0) R packages. The complete code with documentation for validating prognostic genes and conducting correlation analysis materials is available on GitHub: https://github.com/bigbiolab/THCA_COL13A1_COL23A1.
